# Transcriptional profiling of zebrafish identifies host factors controlling susceptibility to *Shigella flexneri*

**DOI:** 10.1242/dmm.050431

**Published:** 2024-01-26

**Authors:** Vincenzo Torraca, Richard J. White, Ian M. Sealy, Maria Mazon-Moya, Gina Duggan, Alexandra R. Willis, Elisabeth M. Busch-Nentwich, Serge Mostowy

**Affiliations:** ^1^ Department of Infection Biology, London School of Hygiene & Tropical Medicine, London WC1E 7HT, UK; ^2^ Section of Microbiology, MRC Centre for Molecular Bacteriology and Infection, Imperial College London, London SW7 2AZ, UK; ^3^ Department of Infectious Diseases, School of Immunology and Microbial Sciences, King's College London, London SE1 9RT, UK; ^4^ School of Life Sciences, University of Westminster, London W1W 6UW, UK; ^5^ Cambridge Institute of Therapeutic Immunology and Infectious Disease, University of Cambridge, Cambridge CB2 0AW, UK; ^6^ School of Biological and Behavioural Sciences, Faculty of Science and Engineering, Queen Mary University of London, London E1 4NS, UK

**Keywords:** Acod1, Gpr84, Host-pathogen, RNA-seq, *Shigella*, Zebrafish

## Abstract

*Shigella flexneri* is a human-adapted pathovar of *Escherichia coli* that can invade the intestinal epithelium, causing inflammation and bacillary dysentery. Although an important human pathogen, the host response to *S. flexneri* has not been fully described. Zebrafish larvae represent a valuable model for studying human infections *in vivo*. Here, we use a *Shigella*-zebrafish infection model to generate mRNA expression profiles of host response to *Shigella* infection at the whole-animal level. Immune response-related processes dominate the signature of early *Shigella* infection (6 h post-infection). Consistent with its clearance from the host, the signature of late *Shigella* infection (24 h post-infection) is significantly changed, and only a small set of immune-related genes remain differentially expressed, including *acod1* and *gpr84*. Using mutant lines generated by ENU, CRISPR mutagenesis and F0 crispants, we show that *acod1-* and *gpr84*-deficient larvae are more susceptible to *Shigella* infection. Together, these results highlight the power of zebrafish to model infection by bacterial pathogens and reveal the mRNA expression of the early (acutely infected) and late (clearing) host response to *Shigella* infection.

## Teaser

**Summary:** The transcriptional signature of zebrafish larvae infected with *Shigella flexneri* identifies *acod1* and *gpr84* as inducible factors that play a significant role in *S. flexneri* infection control.

## INTRODUCTION

The zebrafish has emerged as an important animal model for studying human infection (Gomes and Mostowy, 2020; Stream and Madigan, 2022; Torraca and Mostowy, 2018). Zebrafish larvae are genetically tractable, optically accessible and have a fully functional innate immune system with macrophages and neutrophils that mimic their mammalian counterparts. A wide variety of pathogenic bacteria have been investigated using zebrafish models, providing unprecedented resolution of the cellular response to infection *in vivo*.

*Shigella flexneri* is an important human pathogen and the causative agent of bacillary dysentery. It is highly infectious and its prevalence is highest in tropical/subtropical regions where access to safe drinking water is limited. Mice are naturally resistant to *S. flexneri* infection (Schnupf and Sansonetti, 2019), although new work has shown that NAIP–NLRC4-deficient mice are susceptible to oral *S. flexneri* infection and recapitulate clinical features of shigellosis (Mitchell et al., 2020). A *S. flexneri*-zebrafish infection model has been established, showing that both macrophages and neutrophils are involved in the protective immune response and providing mechanistic insights into interactions between *S. flexneri* and phagocytes (Duggan and Mostowy, 2018; Mostowy et al., 2013; Torraca and Mostowy, 2018). The zebrafish infection model for *S. flexneri* has been shown to successfully recapitulate crucial features of shigellosis, including inflammation and macrophage cell death (Mazon-Moya et al., 2017; Mostowy et al., 2013), and has been valuable in highlighting key roles of virulence factors [e.g. type three secretion system (T3SS) and O-antigen] (Mazon-Moya et al., 2017; Mostowy et al., 2013; Torraca et al., 2019) and cell-autonomous immunity (e.g. autophagy and the septin cytoskeleton) (Mazon-Moya et al., 2017; Mostowy et al., 2013) in host-pathogen interactions.

Here, using our *S. flexneri*-zebrafish infection model, we reveal the mRNA expression of the early (acutely infected) and late (clearing) host response to *S. flexneri* infection at the whole-animal level. We discover a rapid but largely transient activation of immune-related processes in response to sublethal *S. flexneri* infection. Strikingly, only a discrete set of immune genes (including genes encoding the aconitate decarboxylase Acod1 and the G-protein coupled receptor Gpr84) remain differentially expressed at 24 h post-infection (hpi), suggesting an important role for these markers in host survival. Consistent with this, we show that *acod1* and *gpr84* mutations increase susceptibility to *S. flexneri* infection. Comparative studies with expression data from *Shigella*-infected children demonstrate that GPR84 is also induced in humans, highlighting the translational potential of modelling human infection in zebrafish.

## RESULTS

### Whole-animal RNA-seq profiling of *S. flexneri*-infected larvae

**Fig. 1.**
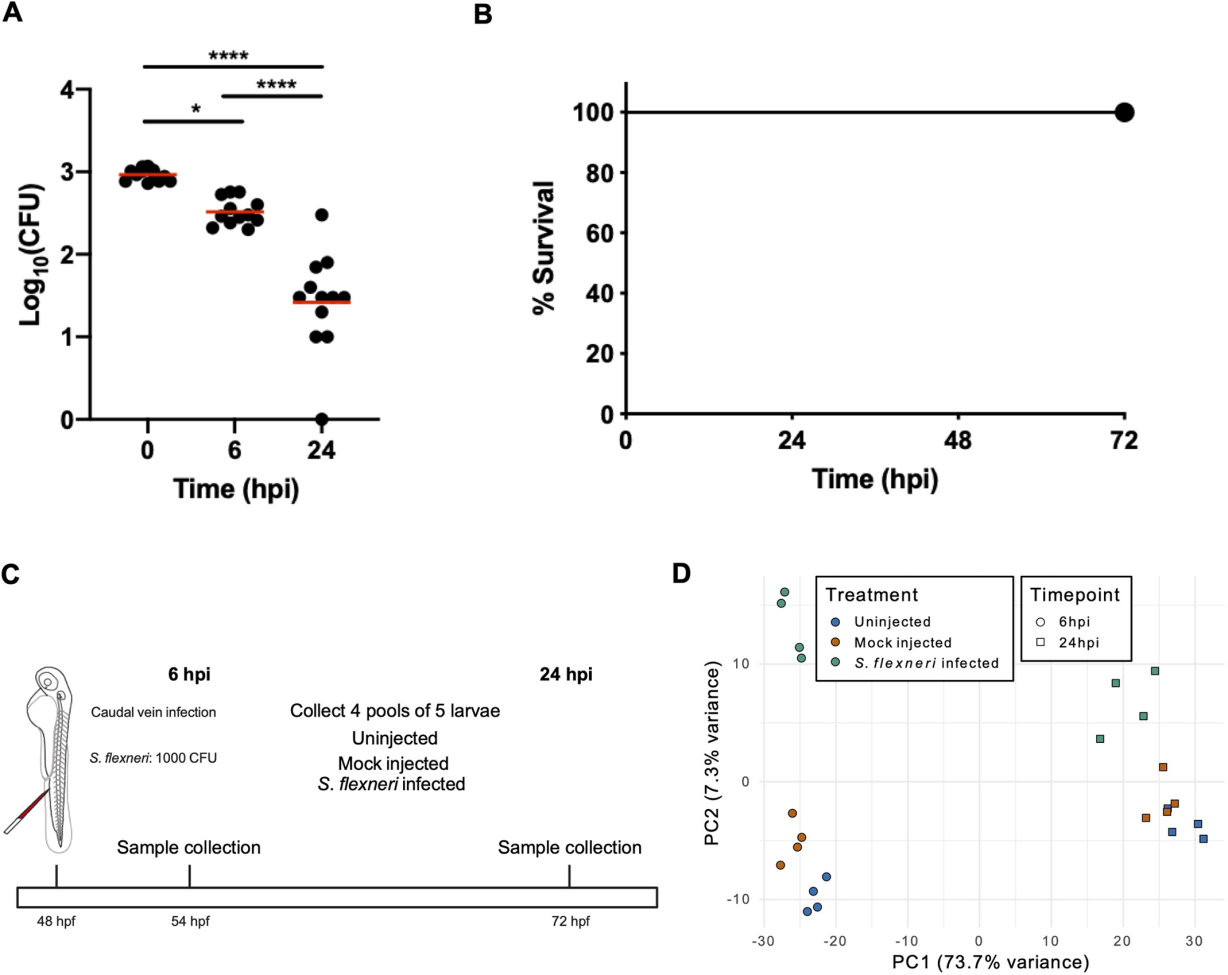
**Experimental design of *Shigella*-zebrafish infection and transcriptomic data collection.** (A,B) Log_10_-transformed CFU counts (A) and survival curves (B) of larvae injected via the caudal vein with 1000 CFU of *S. flexneri*. Injections were performed in 2-day post-fertilisation larvae. *P<0.05; ****P<0.0001 (one-way ANOVA with Tukey's multiple comparisons test). A total of 36 larvae (12 per timepoint) were sacrificed to determine the bacterial load (A), whereas a total of 63 larvae were used for the survival analysis (B). (C) Workflow of the RNA sequencing experiment. Four pools of five embryos injected with 1000 CFU of *S. flexneri* (or mock-injected control) at 24 h post-fertilisation (hpf) were collected at 6 and 24 h post-infection (hpi) for RNA sequencing. Uninfected embryos were also collected at the same timepoints. (D) Principal component analysis (PCA). Regularised log-transformed counts for the 2500 most variable genes across the samples were used in PCA. The first two components are plotted. PC1 separates the samples by timepoint (circle, 6 hpi; square, 24 hpi) and PC2 reflects infection status (blue, uninfected; orange, mock infected; green, *S. flexneri* infected).

To obtain the gene expression response of zebrafish larvae to *S. flexneri* infection, we performed caudal vein injections at 2 days post-fertilisation (dpf). We chose non-lethal infection doses (1000 CFU) by 24 hpi to avoid eliciting non-specific transcriptional responses due to systemic stress. Consistent with this, *S. flexneri* burden decreased over time (Fig. 1A), and zebrafish larvae survived infections for the entire observation period of 72 h (Fig. 1B). We collected four pools of five larvae in three experimental conditions and at two timepoints: uninfected, mock-injected with PBS and *Shigella*-infected groups at 6 hpi and 24 hpi (Fig. 1C). Pooling groups of larvae (as opposed to performing analysis on individual larvae) was adopted to determine the consensus signature of *Shigella* infection in zebrafish larvae, and to mitigate the risk of spurious results due to inter-individual variability. Two time points were chosen to capture the longitudinal change of the host response during the acute infection phase (i.e. 6 hpi) and the pathogen-clearing phase (i.e. 24 hpi). RNA was extracted from all samples and sequencing libraries were produced for RNA-seq.

Principal component analysis (PCA) confirmed that biological replicates clustered according to their condition (Fig. 1D). The developmental stage of larvae explained the biggest principal component, accounting for 73.7% of the variance. The infection state drove the second principal component, responsible for 7.3% of the variance. The largest separation between infected larvae and their corresponding uninfected control was found at 6 hpi, suggesting that the strongest response to infection is at 6 hpi. Overall, these data indicate a sharp distinction between the early acute response (6 hpi) and late clearing response (24 hpi) to infection in *Shigella*-infected larvae.

### Overview of RNA-seq results and gene ontology enrichment analysis

**Fig. 2.**
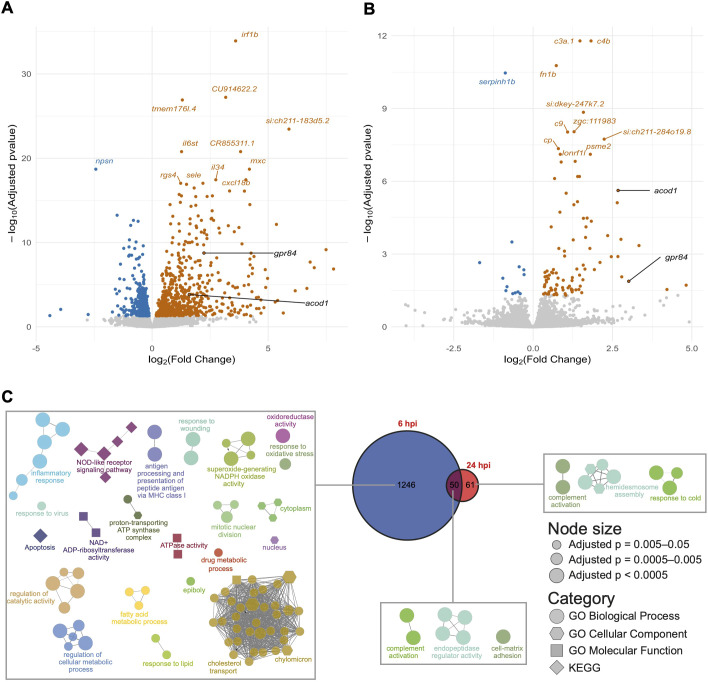
**Analysis of differentially expressed genes in *S. flexneri*-injected embryos.** (A,B) Volcano plots of differentially expressed genes between embryos infected with *S. flexneri* and mock-injected at 6 hpi (A) and 24 hpi (B). Each point represents a gene, -log_10_(adjusted *P* value) is plotted on the *y*-axis and log_2_(fold change) on the *x*-axis. Upregulated genes are coloured in orange and downregulated genes in blue. Genes with the highest -log_10_(adjusted *P* value) are labelled. *gpr84* and *acod1* are highlighted in black as these genes were further pursued for functional characterisation for a role in susceptibility to infection. (C) Gene ontology (GO) term enrichments. Network diagrams of GO term enrichments for genes differentially expressed at 6 hpi only (left), 24 hpi only (right) and both 6 and 24 hpi (middle). Each node in the diagrams represents an enriched GO term, and terms are connected to terms that share annotated genes. This clusters the terms into process-related groups. The Venn diagram shows the numbers of differentially expressed genes at each timepoint and the overlap.

To identify the transcriptional signature of *S. flexneri* infection, we performed differential expression analysis between infected and mock-injected samples for *S. flexneri* infected larvae at 6 and 24 hpi (data available at https://doi.org/10.6084/m9.figshare.20768851.v2). By comparing the two timepoints, we found a much stronger response at 6 hpi, where 1296 zebrafish genes were differentially expressed. Considering that the zebrafish genome GRCz11 has 32,520 gene annotations, this corresponds to 3.98% of all annotated genes. By 24 hpi, expression of these genes returned to levels comparable with that of mock-injected larvae, and the overall number of differentially expressed (DE) genes decreased from 1296 to 111 (corresponding to 0.34% of all annotated genes) (Fig. 2A,B). Additionally, the overlap between the two timepoints (i.e. genes that were consistently DE at both timepoints examined) was only 50 genes (Fig. 2C). Overall, these findings indicate that sublethal infections with *S. flexneri* are characterised by an acute and mostly short-lived variation in gene expression, which subsides as the bacterial load is cleared.

To gain insight into processes transcriptionally regulated in response to *S. flexneri* infection, we performed gene ontology (GO) enrichment analysis. For this, we analysed separately the DE gene lists produced at 6 and 24 hpi. We additionally performed GO analysis for the genes that represented the intersection of the two timepoints (Fig. 2C). At both 6 and 24 hpi, the GO enrichment analysis was dominated by immune-related GO terms, including ‘inflammatory response’, ‘NOD-like receptor signalling pathway’ and ‘complement activation’. In the context of *S. flexneri* infection, the ‘inflammatory response’ might be initiated as a defence mechanism. This response can help to contain and eliminate bacteria, but it can also lead to inflammation and tissue damage if it becomes excessive or chronic. The ‘NOD-like receptor signalling pathway’ plays a pivotal role in the innate immune response of the host, specifically in the detection of intracellular pathogens and sensing of *Shigella* (Girardin et al., 2001). Enrichment of the ‘complement activation’ pathway suggests a systemic immune response is activated against *S. flexneri* infection.

**Fig. 3.**
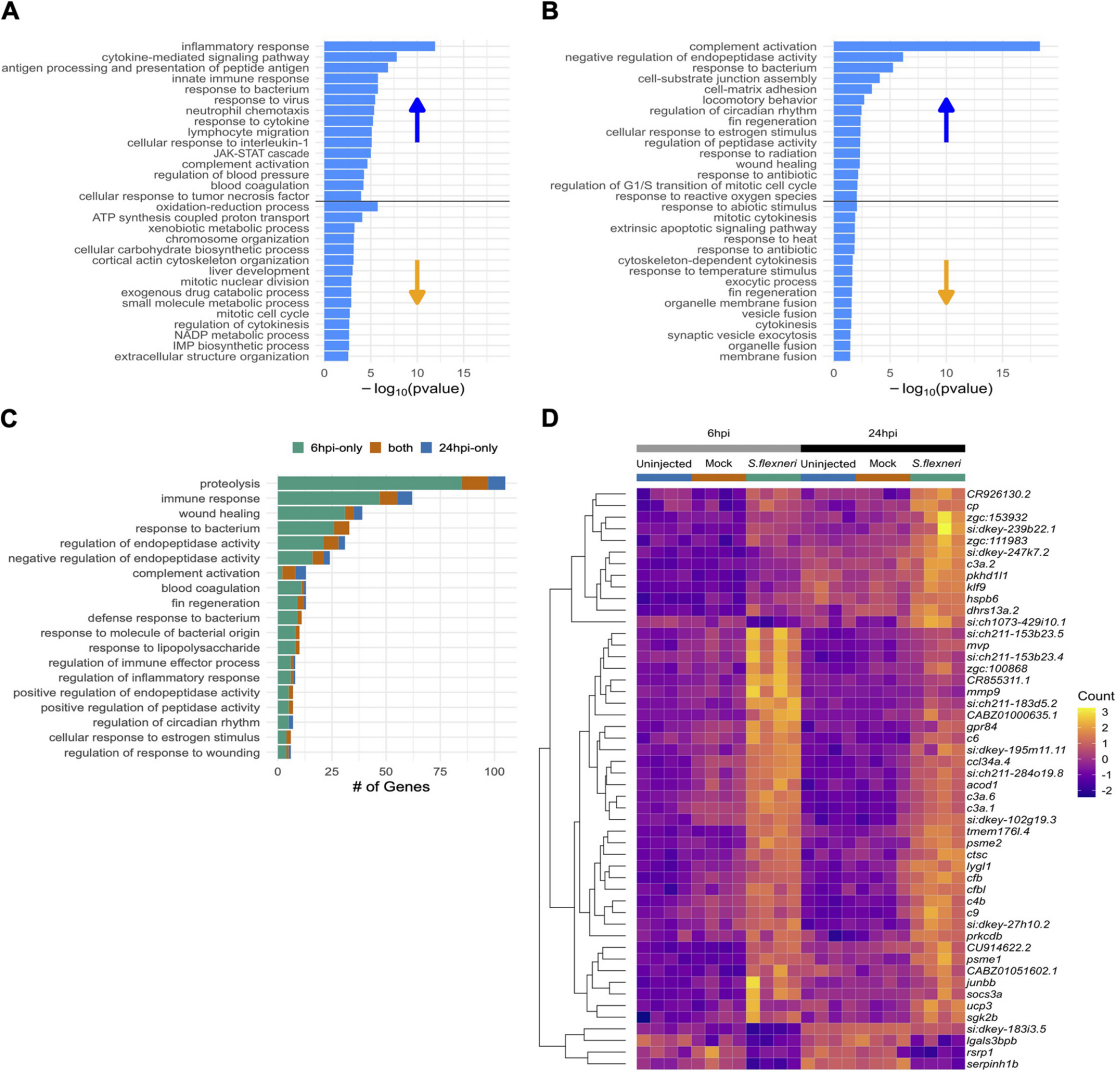
**Gene ontology enrichment analysis of *S. flexneri*-injected embryos.** (A,B) Histogram chart of the top 15 GO terms enriched by either up- or downregulated genes. (A) 6 hpi. (B) 24 hpi. Bars represent -log_10_(*P* value) for the enrichment. Each plot is divided into enrichments caused by upregulated genes (top half) and caused by downregulated genes (bottom half). (C) Bar chart of numbers of genes driving the enrichment of GO terms that are shared between the timepoints. Genes differentially expressed at 6 hpi only are shown in green, those expressed at 24 hpi only are in blue and those expressed at both timepoints are in orange. (D) Heatmap of expression of the 50 genes that are differentially expressed at both 6 and 24 hpi in *S. flexneri* versus uninjected and mock-injected embryos. The colour scale represents normalised counts calculated by DESeq2 that have been mean-centred and scaled by standard deviation for each gene across all the samples.

We next analysed up- and downregulated genes separately (Fig. 3). Genes upregulated at 6 hpi in response to *S. flexneri* led to enrichment of GO terms ‘inflammatory response’ (as above-mentioned), ‘cytokine-mediated signalling pathway’, ‘innate immune response’ and ‘response to bacterium’ (Fig. 3A). The ‘cytokine-mediated signalling pathway’ refers to the cascade of events triggered by cytokines that coordinate the immune response. This pathway can lead to activation of immune cells and regulation of various immune functions, such as inflammation and pathogen clearance. The ‘innate immune response’ represents the first line of defence of the host against a wide variety of pathogens, including *S. flexneri*. This response includes processes such as phagocyte activation, release of antimicrobial peptides and detection of pathogen-associated molecular patterns (PAMPs). The ‘response to bacterium’ encompasses the cellular and molecular processes that occur when a host encounters a bacterium. This could involve recognition of bacteria by the immune system, initiation of immune responses and cellular changes that occur to combat infection. In contrast, enrichment of GO terms for downregulated genes did not include any strictly immune-related terms, but did include ‘cortical actin cytoskeleton organisation’ and ‘regulation of cytokinesis’ (Fig. 3A). These terms indicate that infection leads to a re-organisation of the actin cytoskeleton, which can have implications for cell structure and movement. This finding is in line with the ability of *S. flexneri* to manipulate the host cytoskeleton (Mostowy and Shenoy, 2015).

At 24 hpi, genes upregulated by *S. flexneri* infection were enriched for GO terms ‘complement activation’, ‘response to bacterium’ (both above-mentioned) and ‘wound healing’ (Fig. 3B). Enriched GO terms among significantly downregulated genes included ‘mitotic cytokinesis’/‘cytokinesis’ and ‘cytoskeleton-dependent cytokinesis’ (Fig. 3B). The upregulation of ‘wound healing’ likely highlights an attempt by the host to repair tissue damage caused by infection; this can be important for restoring tissue integrity and function during the clearing phase of infection. Genes downregulated at 24 hpi are related to cytokinesis and cytoskeleton dynamics, indicating a prolonged effect of *S. flexneri* on cytoskeleton remodelling. Despite the overlap of enriched GO terms between 6 hpi and 24 hpi, genes that drove these enrichments were not necessarily the same (Fig. 3C).

Only 50 genes were commonly differentially expressed, irrespective of time point (Fig. 3D). These genes included several complement factors (*cfb*, *c4b*, *c3a.6* and *c3a.1*), *mmp9* (matrix metalloproteinase 9), *lygl1* (lysozyme g-like 1), *psme2* (proteasome activator complex subunit 2), *ccl34a.4* (C-C motif chemokine ligand 34a.4), *acod1* (aconitate decarboxylase 1) and *gpr84* (G-protein coupled receptor 84).

To understand whether transcriptional changes can be attributed to *S. flexneri* virulence, we generated the RNA-seq transcriptome of larvae infected with a *S. flexneri* T3SS mutant (ΔMxiD) at both 6 and 24 hpi (Fig. S1A,B; data available at https://doi.org/10.6084/m9.figshare.20768851.v2). At 6 hpi, we identified 682 genes that are differentially expressed only during infection with wild-type *S. flexneri* (and not during infection with the T3SS mutant), as well as a large number of genes (1908) that were differentially expressed exclusively during infection with the T3SS mutant (and not during wild-type *S. flexneri* infection) (Fig. S1C). At 24 hpi, 59 and 187 genes were uniquely induced during wild-type and T3SS mutant infection, respectively (Fig. S1D). GO enrichment analysis of genes differentially expressed by T3SS mutant infection revealed that few innate immune pathways are enriched, including ‘complement activation’ (at 6 and 24 hpi), ‘chemotaxis’ and ‘response to wounding’ (at 6 hpi) (Fig. S2A).

We analysed up- and downregulated genes separately at 6 and 24 hpi (Fig. S2B,C). Genes upregulated at 6 hpi in response to the T3SS mutant led to enrichment of several GO terms also induced by wild-type infection, including ‘inflammatory response’, ‘neutrophil chemotaxis’, ‘complement activation’, ‘response to bacterium’, ‘JAK-STAT cascade’ and ‘cellular response to Interleukin-1’ (Fig. S2B). This indicates that these pathways are induced by *Shigella* in a T3SS-independent manner. However, several cytokine-related pathways (i.e. ‘cytokine-mediated signalling pathway’, ‘response to cytokine’ and ‘cellular response to tumour necrosis factor’) appeared among the most differentially induced pathways only during infection with wild-type bacteria but not with the T3SS mutant (Fig. 3A, Fig. S2B), indicating that T3SS-mediated virulence might be important to elicit these pathways. Similar to infection with wild-type bacteria, enrichment of GO terms for downregulated genes did not include any strictly immune-related terms (Fig. 3A, Fig. S2B).

At 24 hpi, genes upregulated by T3SS mutant infection are enriched with GO terms including ‘complement activation’ and ‘response to bacterium’, whereas downregulated genes are not enriched for any strictly immune-related term, which is similar to what was observed in response to infection with wild-type bacteria (Fig. 3B, Fig. S2C). Despite overlap of enriched GO terms between infection with wild-type and T3SS mutant, genes driving these enrichments were mostly not the same. Together, whole-animal RNA-seq profiling identified a previously unreported set of markers of *S. flexneri* infection and zebrafish host defence (data available at https://doi.org/10.6084/m9.figshare.20768851.v2).

### Discovery of host factors controlling *S. flexneri* infection

To test roles of selected DE genes, we took advantage of previously published mutants and ENU-induced mutations from the Zebrafish Mutation Project (ZMP) (Kettleborough et al., 2013). We also generated new mutants using CRISPR/Cas9 mutagenesis (Brocal et al., 2016).

**Fig. 4.**
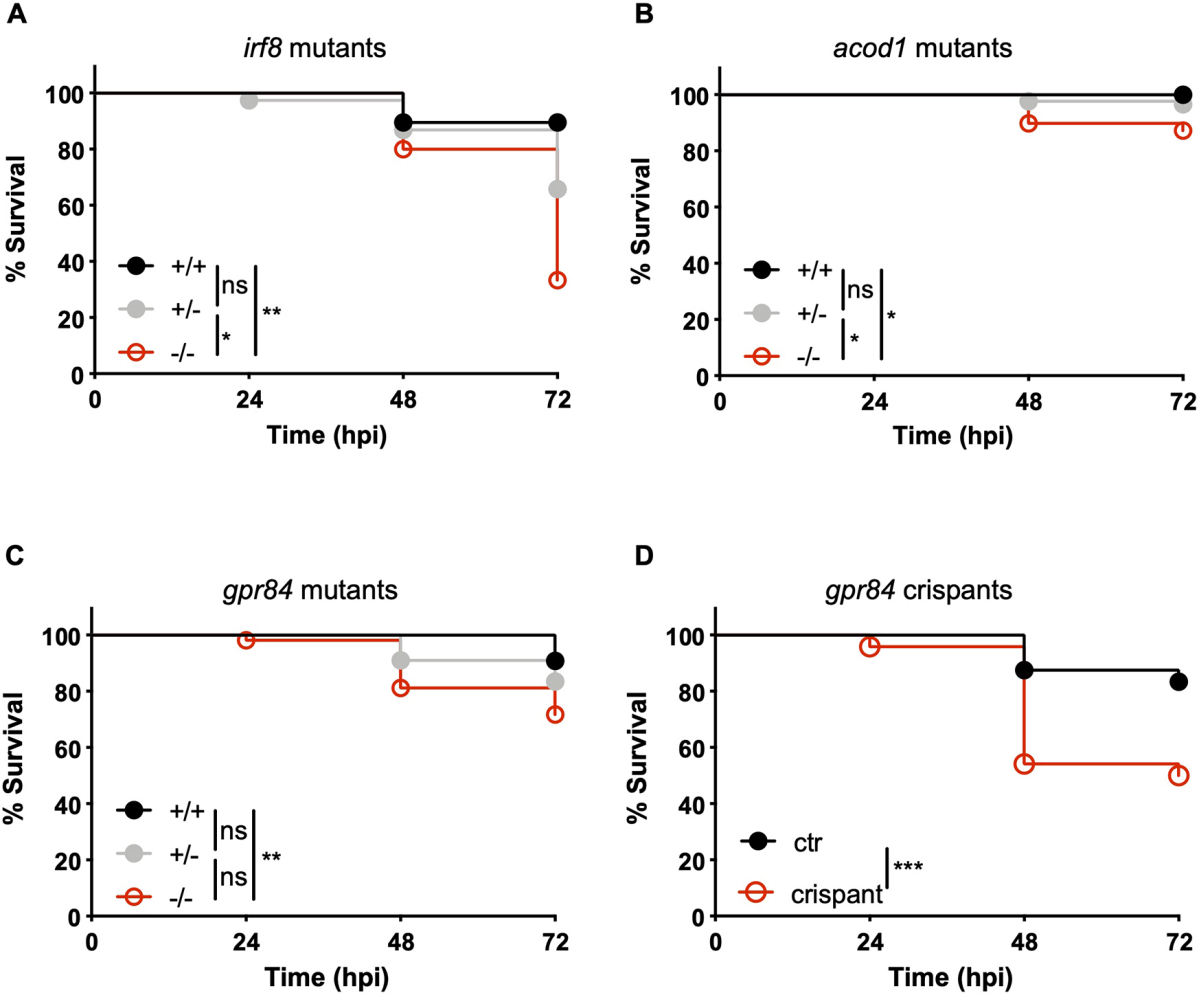
**Functional analysis of zebrafish mutants with susceptibility to infection.** (A-D) Survival curves of larvae derived from incrosses of *irf8* (A), *acod1* (B) or *gpr84* (C) heterozygous carriers of null mutations, and survival curves of *gpr84* crispant larvae or controls (D). Injections were performed in 2-day post-fertilisation larvae via the hindbrain ventricle with 5000-10,000 CFU of *S. flexneri*. For A-C, all larvae were individually genotyped post-mortem or at the end of the experiment. For D, a few randomised larvae were individually genotyped to confirm efficient CRISPR targeting. *P<0.05, **P<0.01; ***P<0.001 [Log-rank (Mantel-Cox) test]. A total of 72 (A, 19 homozygote wild types; 38 heterozygotes; 15 homozygote mutants), 172 (B, 46 homozygote wild-types; 87 heterozygotes; 39 homozygote mutants), 262 (C, 76 homozygote wild-types; 133 heterozygotes; 53 homozygote mutants) or 96 (D, 48 crispants and 48 controls) larvae were used for the survival analyses.

We first established a method to screen for susceptibility phenotypes using the previously published *irf8* mutant line (Shiau et al., 2015). *irf8* mutants have significantly reduced macrophage numbers, and we have previously shown that *irf8* depletion (by morpholino oligonucleotide) and macrophage ablation (by metronidazole treatment in a transgenic line expressing the nitro-reductase enzyme in macrophages) increase susceptibility to *S. flexneri* (Mazon-Moya et al., 2017; Torraca et al., 2019)*.* In line with these observations, a significant difference in survival between homozygous *irf8* mutants and their wild-type siblings could be detected upon infection with *S. flexneri* via the hindbrain ventricle (Fig. 4A). From this, we concluded that survival assays after hindbrain infection represent a high-throughput system with which to study the role of the markers discovered by RNA-seq in controlling susceptibility to *S. flexneri* infection.

Using this experimental design, we explored the role of *acod1* (also known as *irg1*, immunoresponsive gene 1) and *gpr84* (G protein-coupled receptor 84) in the context of *S. flexneri* infection, as these two factors are among the few genes that are consistently induced by *S. flexneri* at both investigated time points. Work has shown that these genes are infection inducible (Recio et al., 2018; Wu et al., 2022), and expressed in innate immune cells in humans and zebrafish (Rougeot et al., 2019) (Fig. S3). Therefore, we reasoned that these genes may play a key role in the innate immune response to *S. flexneri*. Strikingly, homozygous mutants for the *acod1^sa31153^* or the *gpr84^sa10052^* allele are significantly more susceptible to *S. flexneri* infection than their wild-type siblings (Fig. 4B,C). As the *gpr84* mutant was characterised for the first time in this study, its role in controlling *S. flexneri* infection was also confirmed by F0 CRISPR/Cas9 mutagenesis, and *gpr84* crispants phenocopied *gpr84* homozygous mutants (Fig. 4C,D). A higher death rate upon infection in wild-type crispant control larvae (when compared with infection of wild-type larvae that were not injected with crispant control reagents) may be due to minor delays in development provoked by injections and/or handling, as well as direct toxicity of the injected reagents that might increase susceptibility to *Shigella* infection. The role of *acod1* in zebrafish infections has been previously characterised using a *Salmonella* infection model and morpholino knockdowns (Hall et al., 2013), and our phenotype using *S. flexneri* infection is consistent with this work; in this case, we did not consider additional validation by F0 CRISPR to be necessary.

The induction of *acod1* and *gpr84* by *S. flexneri* could have important implications for understanding infection progression. Acod1 is an enzyme involved in the tricarboxylic acid cycle (also known as the citric acid cycle or Krebs cycle) (Wu et al., 2020). It plays a role in conversion of cis-aconitate to itaconate, a metabolite with immunomodulatory properties that can affect the ability of the host to combat infections (Chen et al., 2020; Wu et al., 2022). Gpr84 is a cell surface receptor that can be activated by fatty acids and lipids (Wojciechowicz and Ma'ayan, 2020). When activated, it can trigger intracellular signalling pathways via G-proteins. This can lead to regulation of several immune-related processes, such as phagocytosis, expression of inflammatory mediators and cell migration (Recio et al., 2018; Wojciechowicz and Ma'ayan, 2020).

To explore the translational potential of our study, we compared our RNA-seq expression signature with that reported for *Shigella*-infected children (determined from whole blood RNA-seq analysis) (DeBerg et al., 2018). For this, we applied G:Orth to obtain the list of human orthologous genes for the zebrafish genes that we identified as differentially expressed at 6 and 24 hpi, and compared these with genes differentially expressed in the bloodstream of infected patients. In this case, 12 and four *Shigella*-responsive genes at 6 hpi and 24 hpi were represented in this dataset (Fig. S4); among these genes, *GPR84* (the homologue of zebrafish *gpr84*) was identified. Although the overlap between zebrafish and human data is small (6.5% and 2.2% of genes differentially expressed in human *Shigella* infections are represented in zebrafish datasets at 6 and 24hpi, respectively), there may be several explanations for this. We discovered that response to *S. flexneri* is strongly time dependent and changes rapidly (Fig. 2). Timing of infection in the available human data was not controlled. The patients' dataset is derived from blood samples, whereas our zebrafish data provide the transcriptional signature at the whole-organism level. This allowed us to capture organ-specific responses, such as production of complement factors, which are known to be predominantly liver derived (Zhou et al., 2016). Human data are affected by large interindividual variability. In contrast, our work in zebrafish is highly controlled and performed using clutches of zebrafish siblings to mitigate sources of variability. Finally, lineages of *Shigella* that infect patients are not defined in human datasets. There are four *Shigella* lineages, and over 40 different *Shigella* serotypes (Liu et al., 2008). Instead, in our work, we only used a well-characterised *Shigella* strain (*S. flexneri* 5a, M90T). This is important, considering that key differences in the immune response are elicited by different *Shigella* lineages (Torraca et al., 2019, 2023b).

## DISCUSSION

The zebrafish larva is a powerful non-mammalian vertebrate model that has been instrumental in the study of the innate immune response to bacterial infection (Gomes and Mostowy, 2020; Stream and Madigan, 2022; Torraca and Mostowy, 2018). We have previously used *S. flexneri* infection of zebrafish to study bacterial autophagy (Mostowy et al., 2013), predator-prey interactions (Willis et al., 2016), P1-bacteriophage-mediated immunity (Huan et al., 2023), septin-mediated immunity (Mazon-Moya et al., 2017; Torraca et al., 2023a; Van Ngo et al., 2023), innate immune training (Gomes et al., 2023; Willis et al., 2018), *Shigella* speciation (Torraca et al., 2019) and *Shigella* persistent infection (Torraca et al., 2023b). Here, using RNA-seq, we transcriptionally capture the host response to *S. flexneri* infection over time. We hope this work will serve as a reference for future *in vivo* studies of *S. flexneri* infection. Moreover, it is envisioned that discoveries from genome-wide host RNA signatures of infectious diseases can be used for clinical translation.

*S. flexneri* is a bacterial paradigm used to study the host response to infection (Duggan and Mostowy, 2018; Schnupf and Sansonetti, 2019). Despite intense investigation using various model systems, the host response at the whole-animal level has not been fully described, partly because there is no natural mouse model to study *S. flexneri* infection. To our knowledge, the whole-animal immune response to *S. flexneri* infection has not previously been published in any other vertebrate model, including work using the recently developed inflammasome-deficient mouse model presenting symptoms of shigellosis (Mitchell et al., 2020). The responses of different cell types to *Shigella* have been characterised in human cell lines and isolated primary cells. However, the response of different cell types in the context of a whole organism can be significantly different from what can be detected *in vitro*, as each cell type receives and integrates information and cues from the complex *in vivo* environmental context (i.e. in response to other co-existing cell types). Intercellular control mechanisms exist *in vivo* that are difficult to detect and dissect *in vitro* with individual cell types. Working with whole animals also has the advantage of fewer experimental manipulations that can affect the results. Considering these important factors, the detection of whole organism response to infection has great value and can significantly contribute to understanding the complexity of host-pathogen interactions.

In our experimental conditions testing for survival differences, zebrafish larvae start to die from *S. flexneri* infection at ∼48 hpi. This is expected (Mostowy et al., 2013; Torraca et al., 2019), considering that an incubation period and an initial proliferation of bacteria are required for a wide variety of infectious diseases (including *S. flexneri* infection of humans) before symptoms manifest. However, several genes may be differentially expressed before symptoms become visible, considering that the transcriptional response can be dynamic and rapidly rewired. The genes we highlight in this study (*acod1* and *gpr84*) are induced at 6 hpi and their induction is sustained up to 24 hpi. Although zebrafish do not die at 24 hpi under experimental conditions tested here, we hypothesise that this phase of infection is crucial to deciding the fate of larvae in the subsequent days of infection (i.e. at 48 hpi and beyond).

Our transcriptional profiles suggest a lasting role for *gpr84* and *acod1* during *S. flexneri* infection. Consistent with this, we show that zebrafish mutants in these genes are significantly more susceptible to infection. In the case of *acod1*, similar results were previously published using *Salmonella enterica* Typhimurium (a primary enteric pathogen infecting humans and other animals) (Hall et al., 2013). Although the role of complement factors, chemokines, hydrolytic enzymes and aconitate dehydrogenase have been associated with a variety of infectious and inflammatory processes (Dunkelberger and Song, 2009; Elkington et al., 2005; Murdoch and Finn, 2000; Ragland and Criss, 2017; Sommer et al., 2020; Wu et al., 2022), the precise role of *gpr84* during infection was mostly unknown. Zebrafish Gpr84 protein sequence shares high homology with the human GPR84 protein (Fig. S5), and *in vitro* studies showed that GPR84 is upregulated in macrophages and neutrophils after lipopolysaccharide (LPS) stimulation (Lattin et al., 2008). Additionally, human GPR84 upregulation could be detected in the bloodstream of diarrhoeal children infected with *Shigella* (DeBerg et al., 2018) (Fig. S4). It is next of great interest to understand the precise mechanisms by which *gpr84* is involved in *S. flexneri* infection control.

Mutations of *acod1* and *gpr84* have a modest impact on susceptibility to *S. flexneri* infection. Considering that these genes are infection inducible in innate immune cells (Recio et al., 2018; Wu et al., 2022), they may play a phagocyte-specific and transient role in the host response to *Shigella* that can be difficult to capture at the whole-animal level. For example, *Shigella* is well known to invade epithelial cells (Mostowy et al., 2013), and interactions with these cells are not expected to be impacted by *acod1* and *gpr84* mutations (as these genes are induced primarily in innate immune cells)*.* Finally, considering the breadth of our RNA-seq transcriptome results revealing hundreds of dysregulated genes, more dramatic differences in susceptibility to *Shigella* infection may not be expected from any one single knockdown.

Overexpression by mRNA injection is often applied in zebrafish to complement mutant phenotypes. However, *acod1* and *gpr84* are specific to innate immune cells and mRNA injections in larvae would lead to ubiquitous expression. Considering that *acod1* has a well-described role in regulating the mitochondrial Krebs/tricarboxylic acid cycle (Wu et al., 2020) and that *gpr84* is a member of the G protein-coupled receptor family (widely recognised as master regulators of intracellular signalling) (Yang et al., 2021), overexpression will likely lead to cellular dysfunction and severe aberrations. Therefore, these overexpression experiments were not conducted in the current work.

We observed a more pronounced effect on susceptibility in the *gpr84* crispants compared with the *gpr84* stable mutants (Fig. 4C,D). Several reports have demonstrated that stable mutants are more likely to establish compensatory mechanisms than transiently deficient crispants (Buglo et al., 2020; El-Brolosy and Stainier, 2017; Rossi et al., 2015). Gpr84 is a member of the large protein family of G protein-coupled receptors and, as such, has multiple paralogues. Genetic compensation in the mutant may explain the stronger effect in the *gpr84* crispant experiments.

In the future, it would be interesting to compare the zebrafish response to *S. flexneri* to the response against other pathogens. Although signatures of other pathogens have been described (Lama et al., 2022; Rougeot et al., 2019; Stockhammer et al., 2009; Torraca et al., 2019), the bacterial load, zebrafish strains, embryonic stages, time points used, infection routes and number of pooled larvae per group used by these studies all vary significantly, making it challenging to draw precise conclusions. Instead, we compared our findings with the RNA-seq profile from blood samples of children with shigellosis (DeBerg et al., 2018). This comparison revealed that several markers identified in our work, including *gpr84*, are also identified in response to *S. flexneri* infection in humans and highlights the translational potential of modelling human infection in zebrafish. It will also be informative to compare the immune response to different *Shigella* lineages and/or serotypes, and sort different populations of host cells or individual immune cells from the whole organism to study how different cell types respond to different infections *in vivo*.

## MATERIALS AND METHODS

### Ethics statements

Animal experiments were performed according to the Animals (Scientific Procedures) Act 1986 and approved by the Home Office (Project licenses: PPL P84A89400 and P4E664E3C). All experiments were conducted up to 5 days post-fertilisation.

### Zebrafish handling

Zebrafish lines used here were the wild-type (WT) AB strain. Unless specified otherwise, eggs, embryos and larvae were reared at 28.5°C in Petri dishes containing embryo medium, consisting of 0.5× E2 water supplemented with 0.3 μg/ml Methylene Blue (Sigma-Aldrich). For injections, anaesthesia was obtained with buffered 200 μg/ml tricaine (Sigma-Aldrich) in embryo medium. Protocols comply with standard procedures, as reported at zfin.org.

### Zebrafish mutagenesis

The Sanger mutants used in this study were originally made in the wild-type TL background. The *acod1^sa31153^* allele was created using CRISPR-Cas9 mutagenesis as described previously (Brocal et al., 2016). The CRISPR guide RNA was targeted to chr9:21,836,233-21,836,255 (GRCz11). The guide RNA sequence is 5′-TCCAGGCCAGAGGGTTTAACAGG-3′ (the PAM is underlined). The resulting *acod1^sa31153^* allele is a 35 bp deletion from 21,836,248 to 21,836,282 (i.e. the deletion starts at amino acid 111 and the deleted nucleotide sequence is 5′-TTAACAGGCAGTGTTTCAGCCAGTGCCAGGAGAGC-3′). This mutation leads to a frameshift that is predicted to result in a stop codon after 50 amino acids. Guide RNAs for injection were produced *in vitro* following the method of Hwang et al., (2013). Briefly, overlapping oligonucleotides for the target site (IDT) were annealed and cloned into the pDR274 vector (Addgene 42250) linearised with BsaI (New England Biolabs). Sequence-confirmed vectors were then linearised with DraIII (New England Biolabs), and sgRNA transcripts were generated using the MEGAshortscript T7 Kit (ThermoFisher Scientific). sgRNAs were then DNase treated and precipitated with ammonium acetate and ethanol. Cas9 mRNA was transcribed from linearised pCS2-nls-zCas9-nls plasmid (Addgene 47929) (Jao et al., 2013) using mMessage Machine SP6 kit (ThermoFisher Scientific), DNase treated and purified by phenol-chloroform extraction and ethanol precipitation. RNA concentration was quantified using Qubit spectrophotometer. Approximately 1 nl total volume of 10 ng/μl (sgRNAs) and 200 ng/μl (Cas9 mRNA) was injected into the cell of one-cell stage embryos. Embryos were raised and screened as previously described (Brocal et al., 2016) to isolate carriers. *acod1^sa31153^* was maintained in a mixed AB/TL background.

The mutant allele *gpr84^sa10052^* was generated by ENU mutagenesis (Kettleborough et al., 2013)*.* The introduced point mutation leads to a premature stop codon at amino acid 45. *gpr84^sa10052^* was maintained in the TL background.

### Genotyping

For *acod1* and *gpr84* mutants, genotyping was performed using KASP assays (LGC Biosearch Technologies). For *irf8* mutants and *gpr84* crispants, genotyping was performed using high-resolution melting curve analysis (Garritano et al., 2009; Pagan et al., 2015).

### Bacterial infections

GFP fluorescent or non-fluorescent *S*. *flexneri* M90T (Mostowy et al., 2010; Sansonnetti et al., 1982) and its isogenic mutant ΔMxiD with a defective T3SS apparatus (Allaoui et al., 1993) were used for infections. Bacterial preparation and infection have been described previously (Torraca et al., 2019). For all experiments, 1-2 nl of bacterial suspension (bacterial load is indicated in the individual experiments) or control solution were microinjected in the caudal vein or the hindbrain ventricle (HBV) of 2 days post-fertilisation (dpf) zebrafish larvae, as specified in the individual figure legend. Bacterial enumeration was performed *a posteriori* by mechanical disruption of infected larvae in 0.4% Triton X-100 (Sigma-Aldrich) and plating of serial dilutions onto Congo Red-TSA plates. No significant effect in survival was observed between mutants/crispants and their wild-type siblings when embryos were challenged with mock injections. CFU data were not collected during survival assays as larvae were derived from heterozygote incrosses and were needed for DNA isolation (post-mortem or at the end of the experiment) and genotyping.

### RNA sequencing

RNA was extracted from larvae as described previously (Wali et al., 2022). Briefly, RNA was extracted from a pool size of five fish and four replicates per condition, which balanced experimental feasibility with obtaining sufficient replicates and considering biological variation through pooling. Larvae were disrupted by mechanical lysis in RLT buffer (Qiagen) containing 1 μl of 14.3 M β-mercaptoethanol (Sigma-Aldrich). The lysate was combined with 1.8 volumes of Agencourt RNAClean XP (Beckman Coulter) beads and allowed to bind for 10 min. The plate was applied to a plate magnet (Invitrogen) until the solution cleared and the supernatant was removed without disturbing the beads. This step was followed by washing the beads three times with 70% ethanol. After the last wash, the pellet was allowed to air-dry for 10 min and then resuspended in 50 μl of RNAse-free water. RNA was eluted from the beads by applying the plate to the magnetic rack. Samples were DNase I treated to remove genomic DNA. RNA was quantified using Quant-IT RNA assay (Invitrogen). ERCC spike mix 2 (Ambion) was added to the RNA. Stranded RNA-seq libraries were constructed using the Illumina TruSeq Stranded RNA protocol after treatment with Ribozero. Libraries were pooled and sequenced on Illumina HiSeq 2500 in 75 bp paired-end mode (median 7.3 million reads per sample). Sequence data were deposited in ENA under accession ERP012128. The data were assessed for technical quality (GC content, insert size, proper pairs, etc.) using FASTQC (www.bioinformatics.babraham.ac.uk/projects/fastqc/). Reads for each sample were mapped to the GRCz11 zebrafish genome assembly and counted against the Ensembl version99 annotation using STAR (Dobin et al., 2013). Differential expression analysis was carried out in R (R Core Team, 2019) with DESeq2 (Love et al., 2014) using a cut-off for adjusted *P* values of 0.05.

### GO enrichment analysis

Enrichment of Gene Ontology (GO) terms was carried out using the CytoScape ClueGO app (Bindea et al., 2009). The settings for ClueGO were as follows: a right-sided hypergeometric test (enrichment only) was used with the Bonferroni step-down (Holm–Bonferroni) correction for multiple testing, and terms with corrected *P* values>0.05 were discarded. Enrichment for separate up/down gene lists was carried out using the R topGO package (Alexa et al., 2006).

## Supplementary Material

10.1242/dmm.050431_sup1 Supplementary information

## First Person

This article has an associated First Person interview with the first author of the paper.
