# Supplementary material for: Transcriptional profiling of zebrafish identifies host factors controlling susceptibility to Shigella flexneri
Source: Dis Model Mech. 2024 Jan 26;17(1):dmm050431. doi: 10.1242/dmm.050431 (PMC10846535; doi:10.1242/dmm.050431)
Supplement: Supplementary information [file dmm-17-050431-s1.pdf]

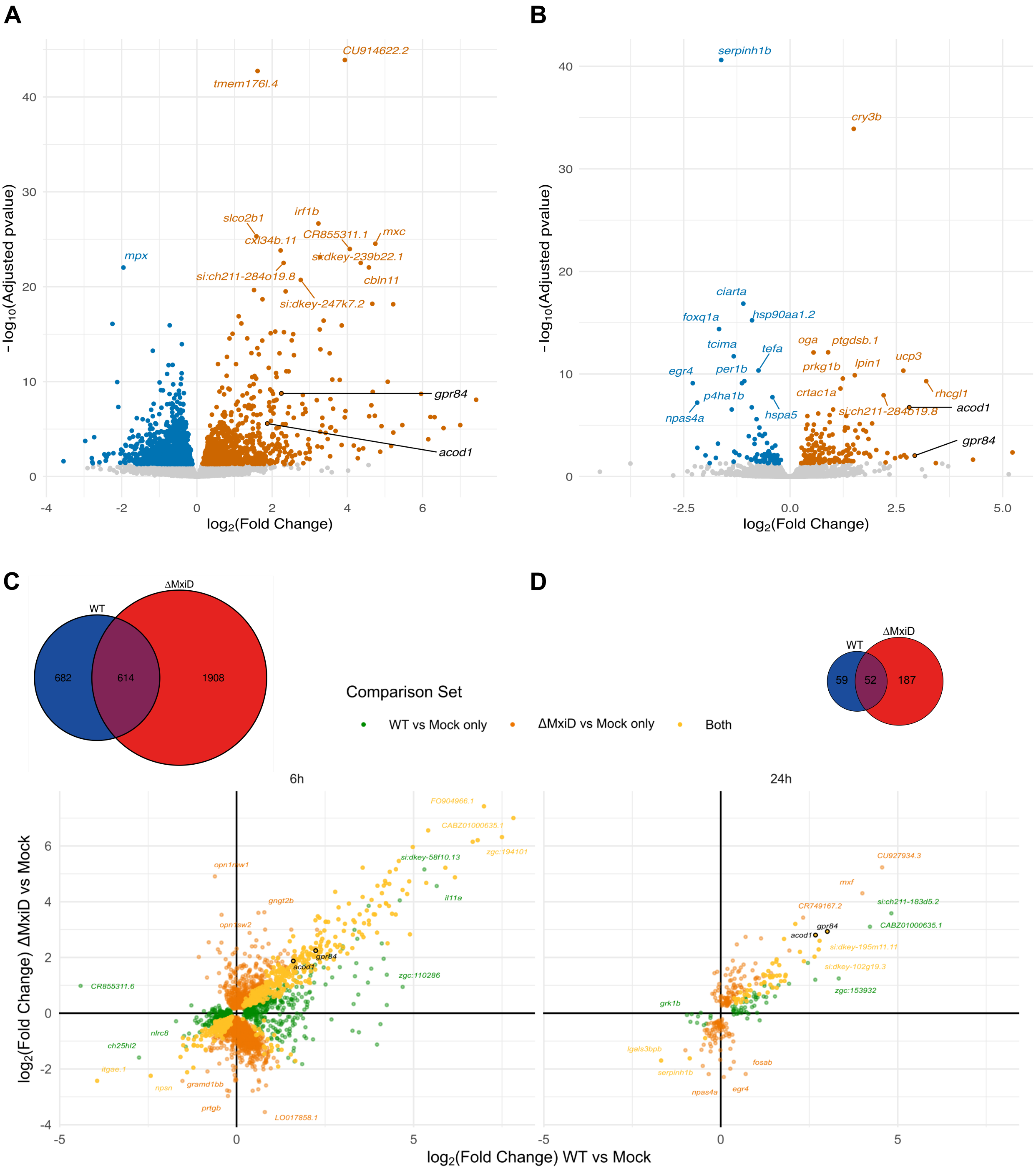

Disease Models & Mechanisms • Supplementary information

Fig. S1. Comparison of differentially expressed genes in larvae infected with WT or ΔMxiD and *S. flexneri*.

A,B. Volcano plots of differentially expressed genes between embryos infected with ΔMxiD *S. flexneri* and mock-injected at 6 hpi (A) and 24 hpi (B). Each point represents a gene, -$\log_{10}$(Adjusted pvalue) is plotted on the y-axis and $\log_2$(Fold Change) on the x-axis. Upregulated genes are coloured in orange and downregulated ones in blue. Genes with the highest -$\log_{10}$(Adjusted pvalue) are labelled.

C,D. Comparison of $\log_2$(Fold Change) in gene expression of ΔMxiD-*Shigella* infected larvae vs Mock and WT-*Shigella* infected larvae vs Mock at 6 hpi (C) and 24 hpi (D). Each point represents a gene. $\log_2$(Fold Change) for ΔMxiD vs Mock is plotted on the y-axis, and $\log_2$(Fold Change) for WT vs Mock is plotted on the x-axis. Only genes significantly differentially expressed in one or the other condition are represented. Genes that were significantly differentially expressed only in ΔMxiD vs Mock are represented in orange. Genes that were significantly differentially expressed only in WT vs Mock are represented in green. Genes that were significantly differentially expressed in both conditions are represented in yellow. Some of these with the highest $\log_2$(Fold Change) are labelled. The complete lists of differentially expressed genes for both ΔMxiD vs Mock and WT vs Mock are available at https://doi.org/10.6084/m9.figshare.20768851.v2

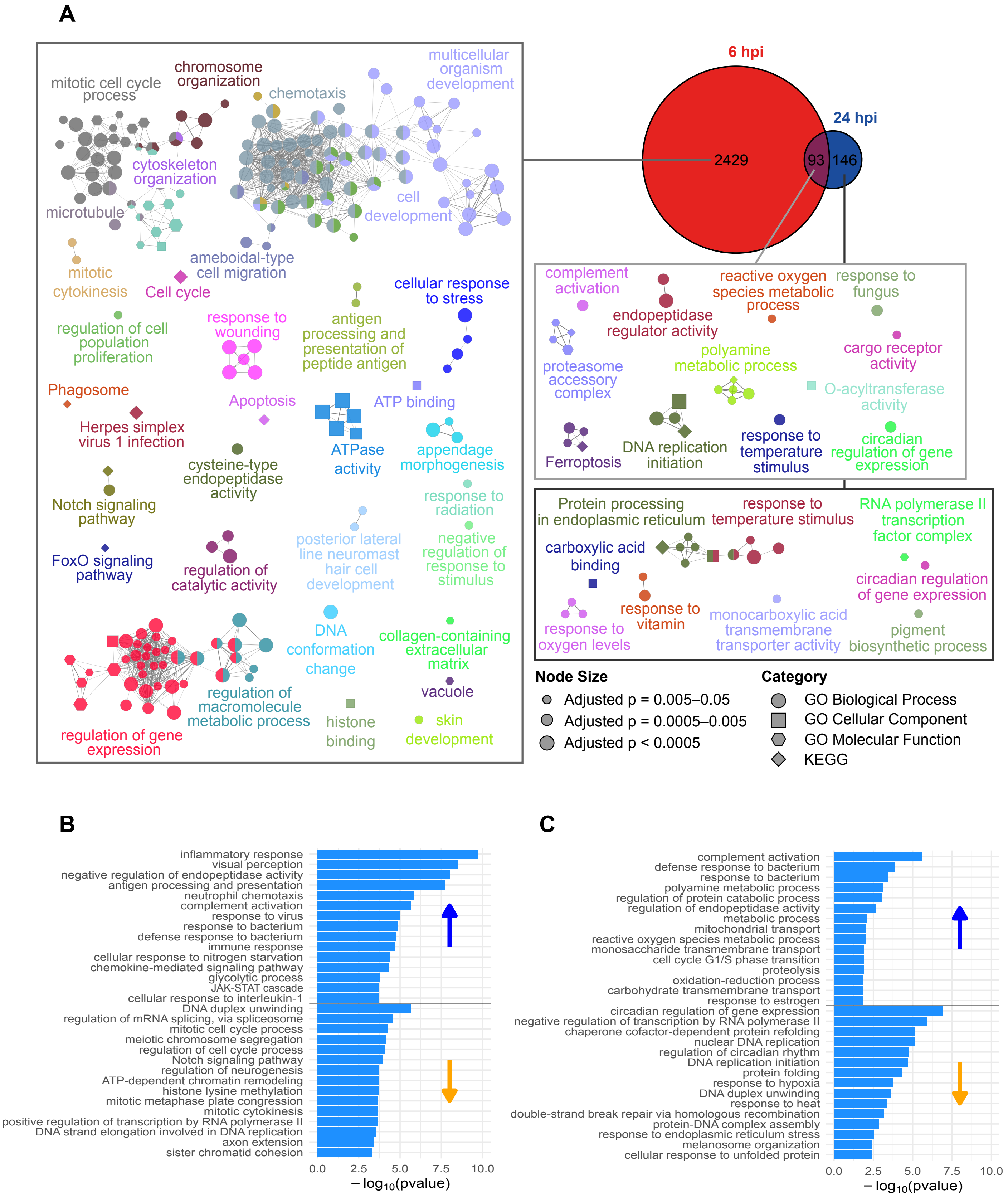

Disease Models & Mechanisms • Supplementary information

Fig. S2. Gene ontology enrichment analysis of ΔMxiD-*S. flexneri* injected embryos. A. Gene Ontology (GO) term enrichments. Network diagrams of GO term enrichments for genes differentially expressed at 6 hpi only (left), 24 hpi only (bottom right) and both 6 and 24 hpi (top right). Each node in the diagrams represents an enriched GO term and terms are connected to terms that share annotated genes. This clusters the terms into process-related groups. The Venn diagram shows the numbers of differentially expressed genes at each timepoint and the overlap.

B,C. Histogram chart of the top 15 GO terms enriched by either up- or downregulated genes. B) 6 hpi. C) 24 hpi. Bars represent $-\log_{10}$(pvalue) for the enrichment. Each plot is divided into enrichments caused by upregulated genes (top half) and caused by downregulated genes (bottom half).

Disease Models & Mechanisms • Supplementary information

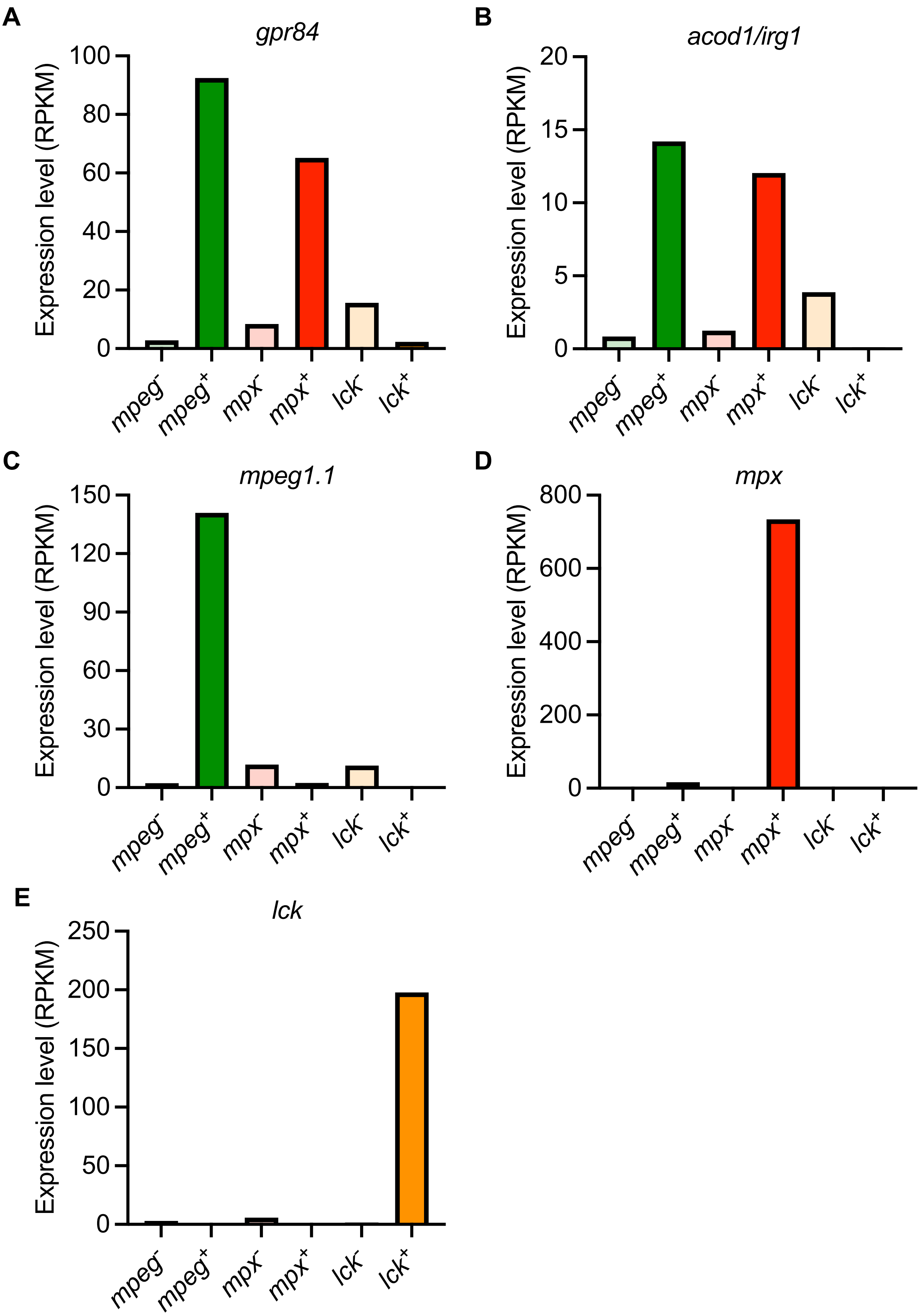

Disease Models & Mechanisms • Supplementary information

Fig. S3. Enrichment of *gpr84* and *acod1* expression in FACS-sorted innate immune cells of zebrafish larvae.

A,E. Expression level of *gpr84* (A), *acod1* (B), *mpeg1* (C, macrophage-specific marker), *mpx* (D, neutrophil-specific marker) and *lck* (E, T cell specific marker) in macrophages (*mpeg1* +), neutrophils (*mpx*+) and maturing T cells (*lck*+) versus their corresponding negative population fractions (*mpeg*-, *mpx*-, and *lck*-, respectively). Cells were FACS-sorted from zebrafish larvae at 5 dpf. Data extracted from Rougeot et al., 2019. *Tg(mpeg1:gal4/ UAS:Kaede*) was used to sort macrophages, *Tg(mpx:GFP)* was used to sort neutrophils, and *Tg(lck:GFP)* was used to sort maturing T cells.

Disease Models & Mechanisms • Supplementary information

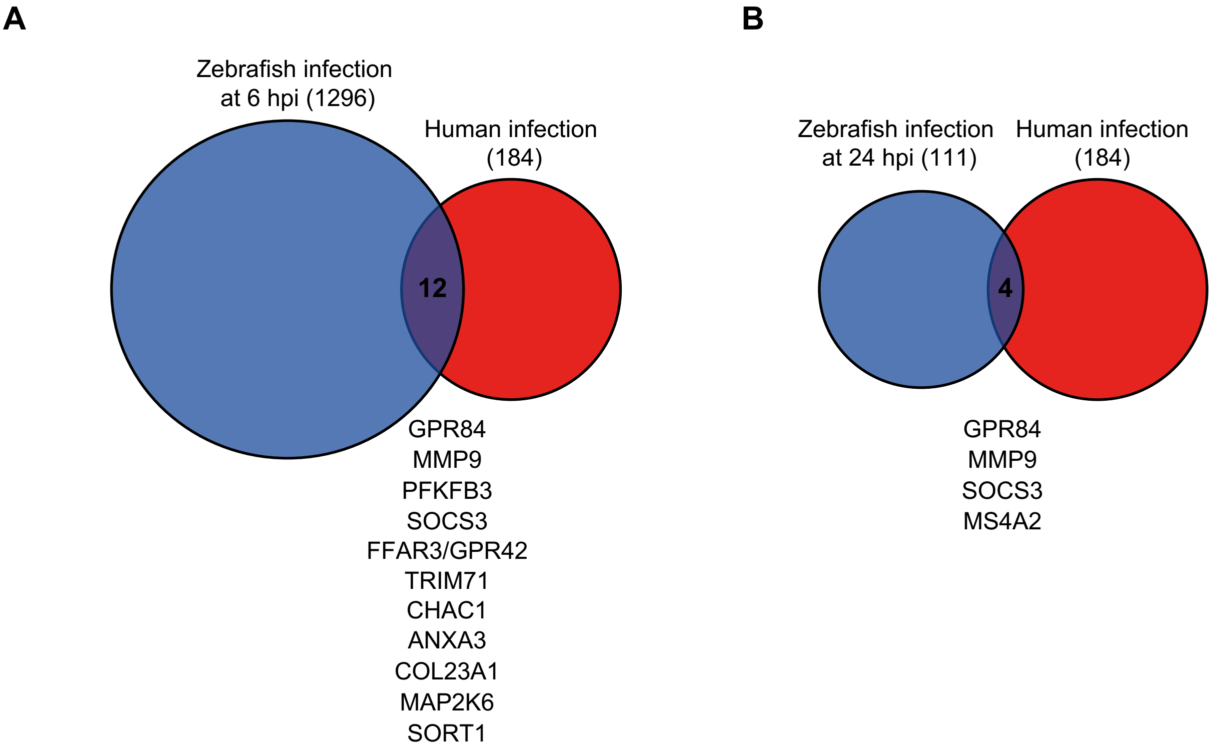

C

Human gene	Human dataset		Zebrafish orthologue	% Sequence Identity	Zebrafish dataset 6hpi		Zebrafish dataset 24hpi	
	Log2FC	p.adj			Log2FC	p.adj	Log2FC	p.adj
**GPR84**	3.29	5.31E-09	gpr84	47.1%	2.23	1.77E-09	3.01	1.32E-02
**MMP9**	2.51	2.11E-07	mmp9	58.1%	3.87	1.44E-10	1.14	2.10E-02
**PFKFB3**	1.54	1.55E-07	pfkfb3	71.6%	0.93	1.66E-02	ns	ns
**SOCS3**	1.53	2.98E-06	socs3a	61.4%	2.65	2.33E-13	1.10	2.47E-02
			socs3b	64.3%	1.48	1.34E-08	ns	ns
**FFAR3**	1.53	5.27E-04	si:ch73-90p23.1	47.1%	2.22	3.22E-02	ns	ns
**GPR42**	1.20	4.28E-03		47.5%			ns	ns
**TRIM71**	1.47	3.40E-03	trim71	75.1%	0.29	3.19E-03	ns	ns
**CHAC1**	1.08	5.38E-03	chac1	57.7%	0.40	3.54E-02	ns	ns
**ANXA3**	1.80	6.50E-07	anxa3a	53.4%	-0.55	3.68E-04	ns	ns
**COL23A1**	1.31	6.70E-04	CABZ01074130.1	38.4%	-0.43	2.58E-03	ns	ns
**MAP2K6**	1.26	1.54E-05	map2k6	79.9%	-0.26	1.28E-02	ns	ns
**SORT1**	1.19	1.59E-06	sort1b	60.3%	-0.31	3.67E-02	ns	ns
**MS4A2**	-1.16	1.51E-03	ms4a17a.8	18.62%	ns	ns	1.44	2.88E-02

Disease Models & Mechanisms • Supplementary information

Fig. S4. Comparison of differentially expressed genes in zebrafish larvae infected with *S. flexneri* and *Shigella*-infected children.

A,B. Differentially expressed genes in *Shigella*-infected zebrafish larvae at 6 hpi (A) and 24 hpi (B), were converted into human orthologues using g:Orth (https://biit.cs.ut.ee/gprofiler/orth) and compared to the list of genes differentially expressed in blood samples from *Shigella*-infected children (DeBerg et al., 2018). Note that for the two human genes GPR42 and FFAR3, g:Orth identifies the same zebrafish orthologue in si:ch73-90p23.1. **C.** Summary table of the genes differentially expressed in both blood samples from *Shigella*-infected children and *Shigella*-infected zebrafish larvae. The table reports human gene symbol, Log2FC and p.adj as in DeBerg et al., 2018, zebrafish orthologue gene symbol, % zebrafish orthologous sequence matching the human sequence, Log2FC and p.adj in zebrafish larvae at 6 and 24 hpi. Log2FC and p.adj highlighted in green indicate upregulated genes, while Log2FC and p.adj highlighted in red indicate downregulated genes.

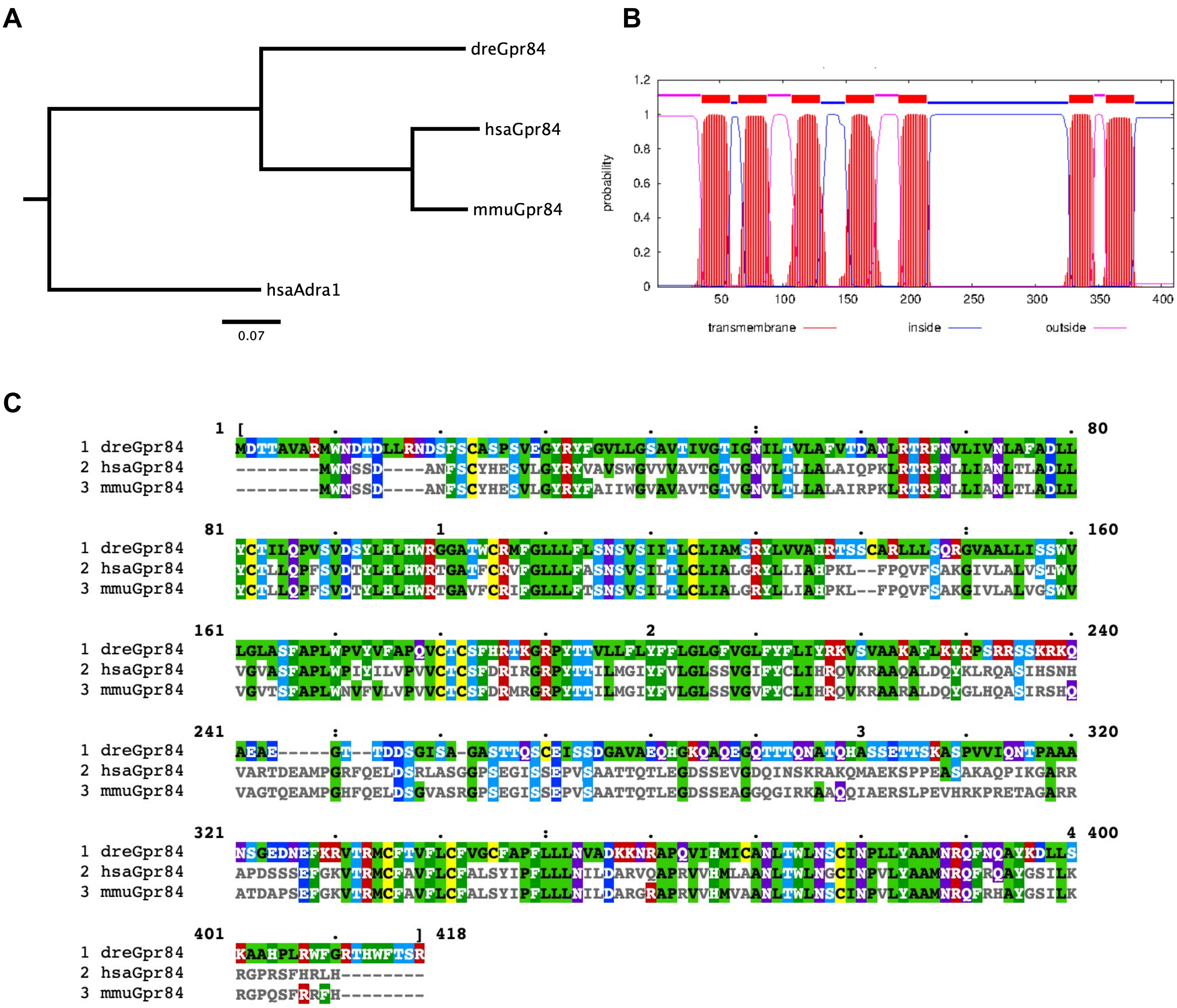
A
dreGpr84
hsaGpr84
mmuGpr84
hsaAdra1
0.07
B
probability
1.2
1
0.8
0.6
0.4
0.2
0
50 100 150 200 250 300 350 400
transmembrane
inside
outside
C
1 [. . . . : . . . 80
1 dreGpr84 MDTTAVARMWNDTDLLRNDSFSCASPSVEGYRYFGVLLGSAVTIVGTIGNILTVLAFVTDANLRTRFNVLIVNLAFADLL
2 hsaGpr84 --------MWNSSD----ANFSCYHESVLGYRYVAVSWGVVVAVTGTVGNVLTLLALAIQPKLRTRFNLLIANLTLADLL
3 mmuGpr84 --------MWNSSD----ANFSCYHESVLGYRYFAIIWGVAVAVTGTVGNVLTLLALAIRPKLRTRFNLLIANLTLADLL
81 . 1 . . . . : . 160
1 dreGpr84 YCTILQPVSVDSYLHLHWRGGATWCRMFGLLLFLSNSVSIITLCLIAMSRYLVVAHRTSSCARLLLSQRGVAALLISSWV
2 hsaGpr84 YCTLLQPFSVDTYLHLHWRTGATFCRVFGLLLFASNSVSILTLCLIALGRYLLIAHPKL--FPQVFSAKGIVLALVSTWV
3 mmuGpr84 YCTLLQPFSVDTYLHLHWRTGAVFCRIFGLLLFTSNSVSILTLCLIALGRYLLIAHPKL--FPQVFSAKGIVLALVGSWV
161 . . . 2 240
1 dreGpr84 LGLASFAPLWPVYVFAPQVCTCSFHRTKGRPYTTVLLFLYFFLGLGFVGLFYFLIYRKVSVAAKAFLKYRPSRRSSKRKQ
2 hsaGpr84 VGVASFAPLWPIYILVPVVCTCSFDRIRGRPYTTILMGIYFVLGLSSVGIFYCLIHRQVKRAAQALDQYKLRQASIHSNH
3 mmuGpr84 VGVTSFAPLWNVFVLVPVVCTCSFDRMRGRPYTTILMGIYFVLGLSSVGVFYCLIHRQVKRAARALDQYGLHQASIRSHQ
241 : . . . . 3 . . 320
1 dreGpr84 AEAE-----GT--TDDSGISA-GASTTQSCEISSDGAVAEQHGKQAQEGQTTTQNATQHASSETTSKASPVVIQNTPAAA
2 hsaGpr84 VARTDEAMPGRFQELDSRLASGGPSEGISSEPVSAATTQTLEGDSSEVGDQINSKRAKQMAEKSPPEASAKAQPIKGARR
3 mmuGpr84 VAGTQEAMPGHFQELDSGVASRGPSEGISSEPVSAATTQTLEGDSSEAGGQGIRKAAQQIAERSLPEVHRKPRETAGARR
321 . . : 4 400
1 dreGpr84 NSGEDNEFKRVTRMCFTVFLCFVGCFAPFLLLNVADKKNRAPQVIHMICANLTWLNSCINPLLYAAMNRQFNQAYKDLLS
2 hsaGpr84 APDSSSEFGKVTRMCFAVFLCFALSYIPFLLLNILDARVQAPRVVHMLAANLTWLNGCINPVLYAAMNRQFRQAYGSILK
3 mmuGpr84 ATDAPSEFGKVTRMCFAVFLCFALSYIPFLLLNILDARGRAPRVVHMVAANLTWLNSCINPVLYAAMNRQFRHAYGSILK
401 .] 418
1 dreGpr84 KAAHPLRWFGRTHWFTSR
2 hsaGpr84 RGPRSFHRLH--------
3 mmuGpr84 RGPQSFRRFH--------

Fig. S5. ***In silico*** **analysis of zebrafish Grp84 and comparison to human and murine Gpr84.**

A. Phylogeny tree for Gpr84, showing clustering of *Danio rerio* (dre) *Mus musculus* (mmu) and *Homo sapiens* (hsa) protein sequences. The protein sequence for human Adra1 (Alpha-1A Adrenergic Receptor) was the closest protein sequence to human Gpr84 within the human genome and was used to root the tree. The phylogeny tree was obtained by ClustalO, using protein sequences for the canonical protein isoform or the predicted principal isoform. The tree was edited and rooted using figtree.

B. Zebrafish gpr84 is predicted to have a 7-loop transmembrane architecture by TMHMM - 2.0, representing the typical architecture of a G-protein coupled receptor.

C. Sequence alignment of *Danio rerio* (dre) *Mus musculus* (mmu) and *Homo sapiens* (hsa) Gpr84 protein sequences. Alignment was obtained by ClustalO (https://www.ebi.ac.uk/Tools/msa/clustalo/) and visualised using MView (https://www.ebi.ac.uk/Tools/msa/mview/).
